# The NICU doula: family-centered support in the neonatal intensive care unit

**DOI:** 10.3389/fped.2026.1757060

**Published:** 2026-04-10

**Authors:** Ifeyinwa V. Asiodu, Celestine Ofori-Parku, Amritha Somasekar, Caryl L. Gay, Kimarie Bugg, Andrea Serano, Lakethia Jones, Jessica Wade, Sarah Verbiest, Paula Bugg-Wrenn

**Affiliations:** 1 Department of Family Health Care Nursing, School of Nursing, University of California, San Francisco, San Francisco, CA, United States; 2 Reaching Our Sisters Everywhere (ROSE), Atlanta, GA, United States; 3 Mighty Little Giants, Los Angeles, CA, United States; 4 Jordan Institute for Families, School of Social Work, University of North Carolina at Chapel Hill, Chapel Hill, NC, United States

**Keywords:** doula care, NICU (neonatal intensive care unit), NICU families, perinatal mental health and support, postpartum care

## Abstract

**Background:**

Neonatal intensive care unit (NICU) admissions are steadily increasing in the United States. Doulas have been identified as a mitigating strategy to address maternal and infant health disparities. Yet, little is known about the experiences of doulas in NICU settings.

**Objectives:**

The purpose of this study was to understand how doulas support postpartum families in the NICU environment.

**Methods:**

A modified critical ethnographic qualitative study informed by the Life Course Perspective was conducted. Data were collected through a structured listening session and online demographic survey. Thematic analysis was used to code the data and identify key themes and subthemes.

**Results:**

A sample of nine individuals participated in the structured listening session. All of the enrolled participants self-identified as women, were currently practicing doulas, and had NICU experience. Many participants expressed personal and professional experiences that informed their decisions to support postpartum families in the NICU environment. Key themes included motivations; barriers and challenges; facilitators and successes; identified training needs; and opportunities for improvement. Doulas also discussed the importance and need of family-centered care in the NICU.

**Conclusion:**

Our findings suggest doulas are currently supporting postpartum families in NICU settings. However, there are several opportunities to improve the NICU environment to be more welcoming of doulas, while also building the capacity of doulas to support the unique needs of postpartum families during a NICU admission. Future research needs to further explore the barriers and facilitators of integrating doulas in NICU settings.

## Introduction

In the United States (US), admission to neonatal intensive care units (NICUs) is on the rise (1). NICUs are specialized hospital units that provide intensive medical care to preterm or critically ill newborns and infants (1). Preterm birth, earlier than 37 weeks gestation, is one of the leading causes of NICU admission (2–6). Preterm birth is associated with low birth weight and very low birth weight (2–6). These conditions contribute to an increased risk of infant mortality and morbidity, respiratory distress, chronic infections, feeding challenges, poor weight gain, and developmental delays (3–6). Other reasons for NICU admission include birth defects, breathing difficulties, infections, and other medical conditions or illnesses (3–6).

Mothers with infants in the NICU often experience significant physical and mental health challenges after birth (7). These challenges include higher rates of anxiety, postpartum depression, and post-traumatic stress disorder (7, 8). NICU mothers also face more chronic diseases, such as hypertension and diabetes, more perinatal complications, and unmet social needs (8). Recent studies have noted NICU mothers often prioritize their infant's needs which may lead to the neglect of their own pressing health issues or deteriorating postpartum health (8).

Between 2016 and 2023, NICU admissions increased from 8.7% to 9.8% of births, reflecting a 13% increase (1). This increase in NICU admission rates was observed across several demographic groups including—maternal age, race and ethnicity, birthweight, gestational age, and geographic location (1). NICU admission rates were most prominent for older mothers (e.g., ≥40 years old), racially minoritized mothers, and mothers living in Delaware, New Jersey, Utah, or New York (1).

With about 10% of all infants born in 2023 admitted to the NICU in the US, we know that an increased number of families are in need of tailored support. Therefore, NICUs need to identify ways to address the unique needs of postpartum families. One potential strategy to better support postpartum families in NICU settings is to utilize doulas (9, 10). However, little is known about the role of doulas in NICU environments and hospital policies and practices that hinder or facilitate doula support among postpartum families with an infant in the NICU.

Doulas are trained professionals who provide emotional and physical support, as well as education to families during pregnancy, birth, and the postpartum period (11). Doulas provide perinatal education, support during labor and delivery, assist with postpartum recovery, lactation, and infant care, and serve as parental advocates in healthcare settings (12, 13). Doulas also help families navigate challenging healthcare environments and support informed decision making processes (11–13). While not medically trained, doulas are important members of the healthcare team who work in collaboration with nurses, physicians, and midwives, and other perinatal professionals to ensure the physical and mental health and safety of pregnant and postpartum families (11). The presence of doulas during the perinatal period has been shown to decrease labor times, lower cesarean births and interventions rates, increase breastfeeding, and reduce stress and anxiety for birthing individuals and families (12, 13).

When we place the overall benefits of doula support in the context of NICU admissions and preterm birth—where racially minoritized families face several systemic and structural factors including racism, inequitable access to quality care, limited lactation support, and poor maternal and infant outcomes (14–16)—then ensuring that postpartum families have access to trained doulas in NICU settings becomes a critical matter of equity and justice. Thus, understanding the experiences of doulas who have supported NICU families is critical to ensuring postpartum families' needs are being met. Yet, data on NICU doula experiences is limited. The purpose of this paper was to understand how doulas can be utilized in NICU settings to support postpartum families.

## Methods

### Research design

A modified critical ethnographic approach, which employed a structured listening session and limited document review, was used. Critical ethnography builds on traditional ethnography by challenging researchers to utilize a critical lens or theoretical framework to explore and analyze how power structures, oppression, and inequities inform social phenomena (17, 18). The phenomenon of interest for this study was doulas in NICU settings. Critical ethnography also moves beyond description to focus on critiquing social injustices and highlighting broader political and economic structures at play; with an ultimate goal of action and sustainable change (17, 18). Due to the exploratory nature of this project, participant observations, prolonged field engagement, and extensive document analysis, hallmarks of critical ethnography were not completed. Additionally, for this project a listening session was defined as a less structured focus group which prioritized relationship-building activities to facilitate greater listening and sharing of perspectives, experiences, and concerns related to providing doula care in NICU settings. The methodology was further informed by the Life Course Perspective (19, 20). The Life Course Perspective includes four elements—timeline, timing, environment, and equity—and seeks to explore lived experiences through a social, structural, historical, and cultural lens (19–21). This framework provides the opportunity to examine and understand how personal histories and life events shape current and future decision-making processes (19–21). The project protocol was approved by the University of California, San Francisco Institutional Review Board. All study participants provided consent prior to the initiation of the structured listening session (e.g., focus group). Data were collected during July 2024. Each participant was remunerated for their time and expertise with a $150 check.

### Participant recruitment and eligibility

Purposive sampling was used to recruit study participants. Participants who self-identified as a doula, were 18 years of age or older, based in the US, and had NICU experience were recruited to participate in the structured listening session. Study recruitment materials were disseminated through social media platforms such as Facebook and Instagram. Information about the study was also shared with community-based organizations, other professional networks, and word of mouth. Prior to being sent the online demographic survey by email, eligibility was confirmed by phone, and consent was obtained through the online survey.

### Data collection

Data were collected using a structured listening session, field notes, and demographic survey. Participant interviews and community participant observations, hallmarks of critical ethnography (17, 18), were not employed for this project. Participants partook in one structured listening session which was convened via Zoom. The structured listening session was guided by an investigator-developed interview guide, see Table 1. The interview guide was created in collaboration with community partners who support NICU families. During the structured listening session, the interview guide was modified to follow emergent themes as they surfaced during the discussion. The structured listening session lasted 2 h, was audio-recorded with permission, and was professionally transcribed verbatim. Rigor was maintained through reflexivity and peer debriefing (22). Reflexivity was maintained through reflective team discussions and memos (22).

**Table 1:** Semi-Structured interview guide.

Domain	Interview Questions
General	
	In what ways can we improve NICU care for families?
	What do you see as barriers to quality care in NICU settings? What barriers have you encountered in the past? Did you seek out any help or assistance for the barriers you experienced?
	What do you see as barriers to doula care in NICU settings?
NICU experiences	
	How did you prepare to support your client or family in the NICU?
	How did having a client and/or family with a baby in the NICU make you feel?
	How were you able to cope with seeing babies in such a vulnerable state?
	How did your support or presence impact the health outcome(s) of the NICU infant?
	How would you describe your relationship with the hospital staff?
NICU resources	
	Please describe the types of strategies you have used to support families coping or dealing with any challenges experienced during the NICU stay.
	What type of resources or support would have been most beneficial to you?
	Based on your experiences, what bright spots (or positive resources or supports) have you observed in the NICU?
Closing	
	If you could share any advice or words of wisdom with family or parents with an infant in the NICU, what would you say?
	Are there any other thoughts you wanted to share with us about your NICU doula experiences? Please tell us more.

### Data analysis

Data analysis was iterative and conducted by IVA, AS, and COP. Thematic analysis was used to examine the structured listening session transcript (23–25). The transcript was first reviewed by COP for accuracy and then reviewed by the data analysis team several times prior to coding. The transcript was coded systematically as concepts related to the doulas’ experiences in NICU environments were identified. Once the coding process was completed, the identified codes were placed into specific categories. The categories were then placed into broader themes. The research team met frequently via Zoom to review and discuss the coding and theme development. The project objectives were reviewed throughout this process to maintain objectivity (23–25). Additionally, documents from key doula and maternal health organizations were reviewed to further inform the analysis (11, 15).

## Results

Of the over 50 individuals who expressed interest in participating in the structured listening session, 31 doulas were screened, and 20 doulas were placed on a waiting list. Due to limited capacity and funding, the research team was not able to follow-up with or screen the doulas placed on the waiting list. However, these doulas were notified via email that enrollment for the project had closed. Of the 31 doulas screened, 16 doulas were eligible and sent the online informed consent form and demographic survey. In total, 15 doulas signed the informed consent form, completed the demographic survey, and were sent the Zoom information for the structured listening session. However, of the 15 doulas who consented, only nine attended the structured listening session. Sample characteristics are summarized in Table 2. All the participants identified as women and doulas. Two-thirds of the study participants, 67% (two-thirds), identified as Black. A majority of the study participants were self-employed, parenting, and identified as heterosexual. Their ages ranged from 29 to 52 years.

**Table 2:** Doula sample characteristics (*N* = 9).

Characteristic	Statistic
Female gender, *n* (%)	9 (100%)
Age, years	
Mean (SD)	36.6 (8.1)
Range	29–52
Race, *n* (%)	
Black or African American	6 (67%)
White	3 (33%)
Status, *n* (%)	
Currently a doula	9 (100%)
Sexual orientation, *n* (%)	
Heterosexual	8 (89%)
Bisexual	1 (11%)
Education, *n* (%)	
Some college or technical school	4 (44%)
College graduate	4 (44%)
Prefer not to answer	1 (11%)
Employment status, *n* (%)	
Self-employed	7 (78%)
Employed	1 (11%)
Retired	1 (11%)
Insurance status, *n* (%)	
Private	5 (56%)
Medicaid	1 (11%)
Private and Medicaid	1 (11%)
Prefer not to answer	2 (22%)
Annual income, *n* (%)	
$0–$25,000	1 (11%)
$25,001–$50,000	5 (56%)
$75,001–$100,000	1 (11%)
Prefer not to answer	2 (22%)
Years of service as a doula	
Mean (SD)	3.2 (2.3)
Range	0.1–7.0
Geographic region of practice	
Midwest (Ohio and Michigan)	5 (56%)
South (Georgia)	4 (44%)

While participants’ doula experiences and environments varied, several common themes were observed throughout the structured listening session. Regardless of educational background, income, or years of doula expertise, each participant discussed their *motivations* for wanting to support NICU families; *barriers and challenges* experienced; *facilitators and successes* observed; *identified training needs*; and *opportunities for improvement* (see Table 3).

**Table 3:** Themes: qualitative findings from structured listening session with doulas.

Themes	Subthemes	Definition
Motivation	Personal lived experiences	Captures a blend of lived experience, clinical knowledge, community need, and commitment to improving emotional, physical, and developmental outcomes for infants and their caregivers that draws doulas to NICU work.
	Professional experiences	
Barriers and challenges	Restricted doula access	Reflects interpersonal, structural, and institutional dynamics that made it difficult for doulas to support families in the NICU.
	Lack of awareness or knowledge	
	Biased attitudes	
	Lack of accountability	
Facilitators and successes	Understanding of the benefit of doulas	Encompasses factors that support successful engagement with NICU families and enhance the doula's ability to provide meaningful care in NICU settings.
	Empathetic care	
	Client-centered care	
Identified training needs	Capacity building	Describes areas where doulas identified additional training needs or structured support that can enhance their abilities to effectively support and serve families in the NICU environment.
	Emotional preparation	
Opportunities for improvement	Improving the NICU experience for postpartum families	Reflects doulas’ recommendations for strengthening NICU systems, including enhancing material and structural supports for postpartum parents, increasing parental involvement in infant care, offering targeted supports for fathers, and developing more formal, integrated models for doula collaboration within NICUs.
	Supporting fathers	

### Motivations

Participants’ descriptions and narratives reflected a blend of lived experience, clinical knowledge, community need, and commitment to improving emotional and developmental outcomes for infants and their caregivers as a source of their motivation for wanting to support families during their NICU stay. For some doulas, having had a child in the NICU themselves was central to their motivation, and they sought to use their experience to support others going through similar circumstances. Noted subthemes included *personal lived experiences and professional experiences*.

“…just having, not only just my own NICU experience but… it seems like there's been an uptick in, NICU births in my area specifically. So, I’ve had quite a few of those recently….” (P1)

“…I've been a NICU parent. And then obviously I've had several clients whose babies were in the NICU. And overall, most people have good experiences, but I have heard some kind of more challenging stories…” (P8)

Other participating doulas connected their motivation to their professional background in clinical care, noting that their firsthand exposure to the NICU setting shaped their understanding of the importance of advocacy and supportive presence.

“I've been a doula for seven years…my background is nursing…moms having doulas as a part of their birthing journey now, and how, efficient and effectively doulas [are]—doulas play a role in [birthing journey] and could play a role in the NICU setting for moms.” (P2)

“I'm also a nursing student, so I'm being able…to see that hands-on [experience], when it comes to NICU. I also have NICU experience personally, as well with my first pregnancy. And, my most recent client had a NICU experience.” (P6)

### Barriers and challenges

The analysis also revealed a recurring theme around the barriers and challenges experienced. Participants described several barriers that made it difficult for doulas to support families in the NICU. These challenges reflected interpersonal, structural, and institutional dynamics that limited the presence and collaborative role of doulas. The participant narratives illustrated several subthemes, including *restricted doula access*, *lack of awareness or knowledge*, *biased attitudes*, and *lack of accountability* at the clinical level, which shaped the environment in which doulas attempted to provide care.

One major subtheme centered on *restricted doula access*, where the presence of doulas was barred in the NICU setting, thus limiting the extent to which doulas were able to support NICU families.

“…they didn't let [me] in the NICU…from what I remember, they were like eight weeks when they hired me. And so, I literally walked beside them the whole pregnancy. And so, I wish that we would’ve been able to bridge that gap.” (P8)

The participant went on to elaborate:

“I wasn't even really allowed to go the day of surgery, which was weird too—like, I understand not being in the OR…like, okay. But I just was very pushed out of the experience when I wanted to offer, you know, care to my clients, and they wanted it, like they wanted to receive that as well.” (P8)

Participants also explained how a *lack of awareness or knowledge among staff* regarding the role and importance of doulas in NICU spaces, led to misunderstandings and dismissive interactions.

“I would say the lack of knowledge of the importance of having a doula in the space, you know…the families are hiring us, they’re paying for us to be there…Just the knowledge of what we are—like our purpose in the space, the birthing space, or even in the NICU…” (P5)

“…because we're not clinical support, we—it's not necessarily in our training. And so that makes nurses nervous when other doulas come in, where when there are doulas that have had a lot of experience or training, then it, you know, they can be super helpful.” (P4)

In addition, doulas described barriers to quality care in the NICU. One such challenge observed by the doulas was a *lack of accountability* for clinicians when families or doulas raised concerns about treatment experiences.

“…holding the nurses accountable when certain issues or concerns are being brought to higher authorities about how the treatment is….so we [doulas] can stop that from patient to patient…I think that nurses need to be held accountable for not providing the best service, especially in that sensitive time.” (P7)

“…And when they're left there alone without any sort of advocate, it's not so much that they're getting bad care on purpose, it's just that, you know, they kind of get the short end of the stick because there's not someone there to help keep the providers accountable…” (P4)

Participants also discussed experiencing or observing various types of biased treatment, including judgment surrounding teen pregnancy, perceived substance abuse, and income level.

“…And I've seen that with some of my moms too. And it [being judged]—and it's something that I think the doctors and nurses need to, you know, kind of think about people as a human. I'm sorry. But, so yeah, I just, I think people need to have more compassion, you know what I mean, in that setting…” (P1)

“My daughter was a teen mother…So, I was able to get her a doula, free of charge, from another organization…So, the nurse, had asked like, how did we get the doula? You know, like, where did she come from? Did we pay for her? I guess cause they seen that we were—we—she received Medicaid, so I know you all couldn't afford, you know, the doula. So, I told them that, you know, they was doing a Medicaid pilot program with this organization and they was partnering doulas with, you know, mothers throughout [state]. And it was free of charge. So, I told the nurse that, but it kind of had me look inside out a little bit, you know, kind of make me just felt, you know, just some type of way…” (P9)

These narratives also highlight that this type of biased treatment was not an isolated incident and reflected a trend in the treatment of NICU families that participants have observed during their careers.

### Facilitators and success

Doulas described several key factors that supported successful engagement with NICU families and enhanced their ability to provide meaningful care in NICU settings. Three subthemes were identified including *understanding of the benefit of doulas*, *empathetic care*, and *client-centered care*. A primary facilitator highlighted by doulas was their ability to offer sustained emotional and relational support that complements the clinical work of NICU staff. Doulas noted that their presence often alleviated the burden on nurses, creating a shared model of care that benefited families.

“…I've had ICU nurses and staff ask like, oh my gosh, can families just hire you for just this? Because like you're a lifesaver. Like we take a lot of work off the nurses, you know, with feeding support, that emotional support, and then they can focus on that safety support.” (P4)

“…like even if they just had a doula from, I don't know, 8:00 to 5:00, Monday, Wednesday, Friday, you know, that could take some of that load off of the nurses. It just, I think it would be a game changer.” (P8)

Another subtheme described by participants was the practice of *empathetic care*, such as engaging family interactions with openness, understanding, and intentional listening.

“…I would just say my biggest preparation is coming in with an open-mind and open heart. Already in that situation, most of the time you don't know if that's [NICU admission] gonna happen until it happens. So, just being open-minded, very personable, using my listening skills, nonverbal cues, those things are, I know they may sound small, but they're very important in the moment because those families need that. And so, that is one of the biggest things that I found that was very beneficial.” (P6)

“…one thing that I did learn before I walked in the room, I always take a breath for myself, and I hold a bubble. So, when I got in that room, I was able to be a listener.” (P7)

Participants’ narratives also described the importance of centering the needs of the client and family, in that moment.

“I prepared by just reminding myself of the type of support that the family initially needed. So…just making sure that I'm using words that are just—that's simple that they can understand..” (P5)

“…it [having a NICU baby] was an emotional toll for her. And just being that support person…for her to be able to call whenever she needed something, you could just tell that she felt like she had an-another family member. Like, I felt like I gained a family member, and she gained me.” (P3)

### Identified training needs

Participants described various training needs that can improve doulas’ abilities to support families in the NICU environment. The noted subthemes were *capacity building* and *emotional preparedness*. Several participants noted the value of receiving training specific to functioning in NICU settings, which would enable them to successfully collaborate with patient care teams. Participants noted that this is a critical need, as integration with the NICU care team is vital to a doula's ability to provide effective care and support NICU families.

“…I think giving doulas some training and some knowledge of like how to operate in the NICU because it's kind of like the OR, it has its own like hierarchy with that nurse at the very top and understanding like what that hierarchy looks like and what that atmosphere looks like and knowing how to operate within it without disrupting it, and while keeping everyone safe.” (P4)

“…I'm in the process of training doulas. And there is a module in my training about NICU and kangaroo care and different things like that…like I can't express highly enough, like how valid that is…” (P8)

Other participants identified training needs based on observations of the unmet needs of NICU families. One such need that was frequently discussed was maternal mental health support, an area that several participants observed as inadequate in the NICU setting. Doulas acknowledged that cross-training in maternal mental health support, or mental health first aid, would improve the extent to which they are able to support NICU families.

“…having the staff and the doula certified in mental health first aid or either maternal mental health because supporting families in the NICU that can be a little sensitive. So, the doula and the staff actually having that mental health background, or something [for] our resources would be a great asset.” (P9)

Participants also reflected on the importance of bereavement training as not all NICU admissions end with a positive outcome such as a discharge home. When a neonatal death occurred, our participants expressed feeling unprepared and wished they had previous training and guidance.

“…also obviously preparing for the worst cause not every NICU baby gets to go home, unfortunately. I mean, I've known doulas who, like their client knows that they're gonna lose their baby at 18 weeks or something, 20 weeks, and the doula doesn't go, they just drop the client because, well, you're not having a birth, why do I need to be there? And that's not okay. But it's because these doulas don't know that they can support and or even how.” (P4)

“… cause we're already like birth and postpartum doulas, but a lot of us don't have the [bereavement] certification when it comes down to [being a] death doula. Like do they have like, you know, trainings or something that you can go and get that certification to help prepare if anything happens, you know, to learn more about?” (P9)

### Opportunities for improvement

In addition to training needs, participants identified several opportunities to strengthen support for families navigating NICU environments. Their reflections described unfulfilled needs in parental inclusion and material and practical support. The noted subthemes presented here are *improving the NICU experience for postpartum families* and *supporting fathers*.

“I just feel like we need more support for these families that can't afford to take that time off work and spend all day with their babies, or maybe they have other kids at home and so they need support with childcare” (P4).

This reflection underscores how economic and caregiving responsibilities can influence families’ ability to engage in the NICU, suggesting opportunities to strengthen wraparound support services. Another participant also described opportunities to create more intentional approaches to engaging families directly in their infant's care.

“But including the parents in that care is something that I have seen that is lacking in that space. So, including the parents, educating them and not just coming and, you know, maneuvering their baby without even the slightest of letting them know, obviously if they're not there and you're doing the care, then that's, you know, warranted. But if they're there and they're watching and they are witnessing, just giving them a little bit more of insight, giving the parents a little bit more of insight on what's going on.” (P5)

Several participants discussed the role of fathers and partners. Often these caregivers are in the NICU pod or waiting room, yet they are rarely engaged or integrated into the care practices.

“…A lot of times fathers are more nervous than the moms are, and they stand in the back. [We need] to pull them up to the forefront and to let them know that, hey, you are a part of everything that's going on here.” (P7)

“…I have found that dads do really well with things to do. There's even some evidence that shows that dads bond better with activity rather than like snuggling…Dads bond really well with a job well done, so that like vasopressin type activity.” (P4)

These narratives highlight how routine clinical workflows may unintentionally limit parental participation and education, suggesting that more deliberate models of family-integrated care could improve bonding, confidence, and continuity after discharge.

## Discussion

Doulas in this exploratory study acknowledged the importance of supporting postpartum families in NICU environments. The findings from this study are timely given the large and growing number of NICU admissions, persistent maternal and infant health disparities, and surging interest in doula care in the US (14–16). Participants’ motivations to support NICU families were driven by personal lived and professional experiences and the rise of NICU births in their communities. This finding aligns with the Campbell-Voytal et al. study of doula motivations (26). Campbell-Voytal and colleagues noted that doulas were motivated by the opportunity to mitigate negative health outcomes and experiences, potential career advancement, and being called to support women during the perinatal period (26). Our study participants also illustrated the lack of engagement of fathers and partners in NICU settings and the need for greater family-centered care practices, inclusive of male-identifying individuals. Paternal involvement has been shown to positively impact their stress and anxiety levels and attachment and bonding between the father-infant dyad (27). Participants also discussed the benefits and importance of doula care in NICU settings, especially as it relates to addressing the emotional needs of postpartum families and serving as a support for the healthcare team. While the impact of doula care has been widely noted across the perinatal period (12, 13), few studies have explored the role and experiences of doulas in NICU settings (9, 10).

Doulas have been identified as an important mitigation strategy to address the rising maternal mortality and morbidity crisis in the US (14–16). As described in the literature, doulas provide non-clinical (e.g., emotional, physical, and educational) support during the perinatal period (11). In addition to the continuous support provided, doulas are known to bridge communication gaps and serve as staunch family advocates (28). Thus, the significance of advocacy and holding the healthcare team accountable for biased care or mistreatment was a salient theme in this study. This central finding reaffirms how doulas can promote health equity, facilitate communication between postpartum families and healthcare team members, and advocate for respectful maternity care (14, 15). These efforts are especially important in NICU settings as families often experience higher levels of stress, anxiety, and fear related to the uncertainty of their infant's condition or prognosis (29, 30).

Moreover, doulas in this study identified the unmet maternal mental health needs of postpartum families with NICU infants. This finding aligns with several studies which have emphasized the need for timely mental health assessment and increased access to culturally-responsive mental health services and resources (31–33). Yet, universal mental health screening of postpartum families is not routinely conducted in most NICU settings (33, 34). The issue of maternal mental health in NICU settings is further exacerbated by social and structural determinants of health such as income, employment, socioeconomic status, food and housing insecurity, and age (34–36). Moreover, experiences with racism, discrimination, and bias also compound maternal mental health experiences of Black NICU mothers and families (34). Integrating doulas in NICU settings, alongside other trained mental health professionals, may help to address and better support the maternal mental health needs of postpartum families (37–39). However, more standardized culturally-responsive doula trainings, which center NICU operations, bereavement and grief, trauma-informed care, maternal mental health, and mental health first aid, are needed (37–40).

Lastly, doulas in this study also described how families face layered demands that can limit their ability to remain present at the bedside. Particularly for postpartum parents, balancing recovery from birth, employment demands, and care of other children can prove very challenging (41, 42). In addition, transportation, accommodation, and food costs associated with NICU visitation remained significant concerns, further impacting parental presence (41, 42). Perinatal doula support has been shown to help disrupt and potentially mitigate the negative effects of social determinants of health (43, 44) and experiences of racism (45, 46). Hence, systemic and structural-level strategies and interventions, including greater integration of doulas in NICU settings are needed to properly meet the unique needs of postpartum families.

### Recommendations

Given the current state of maternal and infant health outcomes in the US, there are several opportunities to improve care for NICU families. One of those potential improvements is the implementation and utilization of doulas in NICU environments. Using our modified critical ethnographic approach and the Life Course Perspective, we developed recommendations grounded in both our data and the existing literature. Both standpoints call on us to move beyond an individual-level focus and instead consider the broader systems and structures at play. Through the presented recommendations, we urge healthcare systems, clinical staff and providers, doulas, and NICU families to engage in activities which can facilitate and build capacity within existing systems and create new initiatives where infrastructure to support NICU doulas is limited or nonexistent.

Strategies to Increase Utilization of Doulas in NICU Environments

- Develop, adopt, and integrate policies that support doula care in the NICU.
- Increase funding for NICU doula programs.
- Implement tracking and monitoring systems to assess the utilization and impact of services provided.
- Advocate for the expansion of insurance coverage for NICU doula services.

Strategies to Ensure Equitable Access to Doulas in NICU Environments

- Uplift programs and initiatives aimed at providing NICU families with guaranteed income during the NICU stay.
- Ensure that all clinical staff and providers receive education on how systemic and structural racism impacts health outcomes in NICU environments.
- Develop actionable measures to address and dismantle systemic and structural racism in NICU environments.
- Establish equitable systems to improve awareness and access to doula services for all families, especially those most burdened by higher rates of preterm birth or NICU admission.

Strategies to Increase Education about the Role of Doulas and Training Opportunities in NICU Environments

- Provide evidence-based education about the role of doulas, their scope of practice, and best practices for integrating doula care in clinical environments to NICU staff and providers.
- Offer regular networking and relationship-building opportunities between community-based doulas and NICU staff and providers.
- Ensure NICU doulas receive culturally responsive perinatal mental health education, bereavement and grief preparation, and trauma-informed care training and that this content is integrated into doula training curricula.

Strategies to Support Research and Innovation related to the Utilization of Doulas in NICU Environments

- Increase funding for research on the role, experiences, and impact of doula care in NICU settings.
- Ensure a wide range of methodological approaches, including qualitative research, mixed methods, observational studies, randomized clinical trials, implementation science, systematic and scoping reviews, and meta-analyses are employed.
- Build infrastructure to ensure ethical collaborations between community-based doula organizations, academic institutions, and healthcare and public health systems to increase knowledge generation and translation of findings.

## Limitations

The experiences shared in this paper were from a small sample of US-based, practicing birth and postpartum doulas. Only one, structured listening session was held on this topic. Thus, study findings are not intended to be generalizable. However, generalizability is not the goal of qualitative inquiry. Additional research specifically focused on the lived experiences of doulas who support postpartum families in NICU settings and barriers and facilitators to incorporating doula care in NICU settings are needed.

## Conclusion

The positive benefits of doula support during the perinatal period are well established. It is important to understand how doulas can support postpartum families in NICU settings. Limited research, qualitative or quantitative, has been conducted to explore the barriers and facilitators of integrating doulas into the NICU environment. Greater efforts are needed to increase postpartum families’ access to doula care, raise awareness and knowledge of the doula role among NICU healthcare team members, and build doula capacity through targeted trainings. A lack of NICU doulas is a missed opportunity to improve infant and family health outcomes. Future research is needed to develop culturally responsive NICU doula training programs and interventions, which are responsive to family needs and reflect guidance from communities most impacted by poor maternal and infant health disparities.

## Data Availability

The raw data supporting the conclusions of this article will be made available by the authors, without undue reservation.
